# A Service User Perspective Informing the Role of Occupational Therapy in School Transition Practice for High School Learners with TBI: An African Perspective

**DOI:** 10.1155/2019/1201689

**Published:** 2019-08-01

**Authors:** Lee-Ann Jacobs-Nzuzi Khuabi, Estelle Swart, Mogammad Shaheed Soeker

**Affiliations:** ^1^Division of Occupational Therapy, Stellenbosch University, Cape Town 7500, South Africa; ^2^Department of Educational Psychology, Stellenbosch University, Stellenbosch 7602, South Africa; ^3^Department of Occupational Therapy, University of the Western Cape, Cape Town 7500, South Africa

## Abstract

**Background:**

In the South African context, there are no specific guidelines regarding how to prepare and support adolescents for the transition from a health care to a high school setting post TBI. This raises questions about the relevance and responsiveness of the current transition practices in occupational therapy in terms of adequately preparing and supporting these adolescents to participate in school and hence exercise their right to a quality education.

**Method:**

This study explored adolescents and other key role players' perspectives on and experiences of the high school transition (i.e., school reentry and continued school participation) post TBI. It was anticipated that this would provide an increased understanding of the enablers and barriers to high school reentry and participation post TBI. This served as a basis to explore the main aim of this study which was to help occupational therapists identify where efforts in terms of service delivery are needed. This study was situated in the interpretivist qualitative paradigm and used a multicase study design, which included semistructured interviews with eight adolescent learners with TBI, their primary caregivers, teachers, and principals as well as observations and documentation review.

**Results:**

This paper will focus on a central theme in the research, namely, the nature and extent of support needed to facilitate the high school transition of adolescents with TBI within a developing context. Similar to the findings of studies conducted in developed contexts, participants highlighted that they felt that adolescents need support at various stages of the school transition. Participants further alluded to support that should be collaborative, coordinated, flexible, and monitored to ensure it is relevant and responsive to these adolescents' changing needs.

**Conclusion:**

The study findings conclude that occupational therapists have a crucial role in fostering an enabling environment (directly and indirectly) through fulfilling various roles including that of a facilitator, intermediary, coach, collaborator, supporter, and advocator.

## 1. Introduction

Traumatic brain injury (TBI) is described as an injury to the brain caused by an external mechanical force, resulting in a decreased or altered state of consciousness [1]. South Africa accounts for 89 000 of the 10 million people affected by TBI globally per year [2]. There are peaks identified in TBI incidence throughout the life span. Mid to late adolescence is a life phase where TBI often occurs [3, 4]. Adolescence is a time for tremendous growth and potential as the young person acquires new skills and undergoes many new experiences. However, this is also a time of adjustment given the physical, psychosocial, and moral changes that adolescents experience as they explore who they are and where they fit in, in life [5–7]. This adjustment to the changes associated with adolescence is often compounded by changes in abilities, skills, and roles brought about by the onset of the TBI. This personal adjustment together with environmental barriers may restrict the adolescent's participation in valued occupations, including high school participation.

School participation refers to “those activities needed for being a learner and participating in the learning environment” ([8], p.S20). It includes engagement in the following: (i) academic (e.g., writing, reading, and math); (ii) nonacademic school-related activities (e.g., break time and self-help tasks); (iii) extracurricular (e.g., sports and clubs); and (iv) prevocational activities. School participation is integral in providing a sense of purpose and structure within learners' lives. Positive school experiences allow opportunities for socialization and a sense of connectivity which contributes to a positive sense of self [5, 7, 9]. Given this value of school, high school participation is hence often a priority goal that adolescents identify as part of their occupational therapy intervention following the onset of a newly acquired disability such as a TBI.

The South African Department of Education recognizes occupational therapists as one of the “duly registered health professionals specifically trained to assist learners who experience neurological barriers to learning” ([10], Form DBE 126, p.2). However, there is a lack of clear and practical implementation guidelines in South African policy and strategic guideline documents on how occupational therapists could prepare and support a learner for the school transition following the onset of a new disability such as a TBI. One could argue this has contributed to inconsistencies in terms of school transition practices amongst occupational therapists observed in the South African context. This raises questions about the relevance and responsiveness of current school transition practice, in occupational therapy, in terms of adequately preparing and supporting these learners to transition and participate in school.

Furthermore, the existing body of literature that informs recommendations on practices pertaining to the support and services required to facilitate the school transition post TBI primarily includes recommendations devised in developed contexts (i.e., contexts characterized by their advanced economy, technology, and infrastructure). These recommendations include well-timed planning, consistent communication, and a collaborative team approach [7, 11, 12]. Whilst these recommendations may serve as a good starting point for occupational therapists in South Africa, it cannot be assumed that these recommendations are directly transferrable to a developing context such as South Africa given the differences in terms of geographical area, culture, socioeconomic status, policy, legislation, and hence the funding models that provide access to rehabilitative and educational related services to learners post TBI [5]. Furthermore, these recommendations are primarily focused on the “observers' perspectives,” i.e., the perspectives of parents, educators, and the treating clinicians of adolescents with TBI ([5]:3).

Very few studies explored the “insider” perspective, i.e., the perspectives on and the experiences of school reentry and participation of the adolescents themselves [5, 7, 13–16]. There is thus a need for further research which seeks to include the perspectives and experiences of the main beneficiary of such school transition and support services, i.e., adolescent learners with TBI. This is crucial as for occupational therapists to render relevant and responsive services they require an increased understanding of the support needs of these learners with TBI. This study therefore sought to explore the perspectives on and experiences of high school learners and their relevant stakeholders regarding school reentry and school participation post TBI within a developing context. It was envisioned that this would illuminate the enablers and barriers to school reentry and school participation of adolescent high school learners post TBI and facilitate an increased understanding of these learners' support needs. This served as a basis to explore the main aim of this study which was to assist occupational therapists to identify where efforts in terms of service delivery are needed and their role(s) in response to providing the relevant support.

## 2. Methods

### 2.1. Research Design

The research approach was situated in the interpretivist (constructivist) paradigm, which seeks to make sense of human experience from the insiders' perspectives, using qualitative methods [17]. The design strategy for this inquiry included a qualitative multicase study (i.e., collective/cross-case study). Merriam and Tisdell ([17], p.38) define a case study as an in-depth description and analysis of a case/“object of the study” (within a specific context) around which there are boundaries. The case study design strategy has been selected as it allows for a focus on a particular phenomenon (i.e., high school transition following TBI), in-depth, holistic descriptions, and interpretation within context using multiple sources of information [17, 18].

### 2.2. Participants

The participants were purposively selected (see Table 1 for the selection criteria).

The final sample size was determined by data saturation and included eight adolescent high school learners with TBI (see Table 2 for the demographic description of learners). The sample further included each of their primary caregivers (i.e., biological parent, legal guardian, or grandparent), a teacher, and the principal of the high school that they attended.

### 2.3. Data Collection

Semistructured interviews, with the use of interview guides, were used as the primary data collection method. Semistructured interviews allowed flexibility in terms of the wording and ordering of questions thus allowing the researcher to adapt within the context of the interview [17]. The questions focused on participants' perspectives and experiences of high school reentry and school participation post TBI (see Appendix A (available here) for an example of an interview guide). Observation and documentation review were used as secondary data sources. Observations were semistructured and included what the researcher felt and saw [17]. Examples of observations included the physical setting, people, and their roles in the interviewing context (i.e., school/home). The documents that were analyzed included learners' medical and school records. The documents served to confirm the selection criterion pertaining to the baseline level of cognitive and language functioning a learner needed to participate in the interview. Documents were further used to provide a biographical description of each learner in accordance with the importance of contextualization for a case study and served to supplement other data generation methods [19].

### 2.4. Data Collection Procedure

Following approval from the Health Research Ethics Committee (HREC), Stellenbosch University (#: S15/03/055), and the Departments of Health and Education, gatekeepers were used to gain entry to a site [20]. Gatekeepers included staff members (mainly occupational therapists and physiotherapists) at various health facilities and within the Department of Education who initially identified and approached possible participants who met the selection criteria. They shared the objectives of the study firstly with the primary caregiver and then with the learner with TBI (i.e., a minor) and asked if they were interested in participating in the study. They were further asked for permission for their contact numbers to be given to the researcher to make initial contact. The researcher obtained consent from the primary caregiver and assent from the learner to participate in the study. Learners provided the name of the principal of their school and a teacher who they felt knew them the best and was part of their school transition post TBI. Thereafter, the principal and the teacher were contacted, the study objectives were explained, and consent was obtained. All interviews were conducted at a date and time that were convenient for the participants.

A preliminary meeting (within the learner's home) was conducted for learners to familiarize themselves with the procedures and to help establish rapport (especially in adolescents who were anxious). On another day, this was followed by one 60 to 90-minute interview or two 30 to 45-minute interviews with the learner, dependent on the learner's levels of fatigue and concentration. These interviews were conducted either in the learner's home or in a private area at the learner's school. A 60 to 90-minute interview was conducted with each of the learner's primary caregivers, teacher, and principals. Teachers and principals were interviewed on the school premises, and all primary caregivers opted to be interviewed at their homes. Post interview debriefing occurred, thus allowing the participants to provide feedback about the research process and for the researcher to clarify observations made during the interview [18].

Interviews were audio recorded with consent from all participants [21]. Field notes complemented the interview data and served to record the researcher's semistructured observations. These recordings were completed directly after the interviews whilst observations and impressions were recent enough for the researcher to recall. The verification of the transcripts included returning it to the participants and requesting them to verify the text.

To enhance trustworthiness and rigor, Lincoln and Guba's Model of Trustworthiness for Qualitative Research ([22]:290, [23]) was applied. The four criteria of credibility, dependability, transferability, and confirmability were strengthened. Credibility was improved through the use of strategies such as data triangulation (i.e., data were generated from multiple participants with multiple perspectives and through the use of multiple data collection methods, by including interviews, observations, and document analysis), reflexivity (i.e., the use of field notes), peer examination (the guidance from supervisors who are experts in the research design), member checking, and structural coherence (i.e., using study objectives to remain focused on the research process) [17]. Transferability was strengthened through dense descriptions of the context, participants, and findings with evidence presented in the form of quotes from participant interviews, field notes, and documents [17]. Specific criteria to strengthen dependability included an audit trail (i.e., the use of an extensive literature review, triangulation, verbatim transcription of interview, member checking, dense descriptions of the theoretical, methodological, and analytical processes, and peer examination) [17].

### 2.5. Data Analysis

The data set consisted of transcribed interviews, field notes, and documents. Data were analyzed using an inductive and comparative analytic strategy. The constant comparative method was used at two stages: (i) within case where each case was “treated as a comprehensive case” and (ii) across cases resulting in a “unified description” across cases ([17], p.234).

The steps undertaken in analyzing the data will be presented as outlined by Merriam and Tisdell ([17], p.204-226) and are presented in Table 3.

### 2.6. Findings

The research data yielded 5 themes, and these included the following:

1. “Change in former sense of self”: this refers to the changes the learners' experience in functional abilities, skills, and roles (specifically the role of the learners) which ultimately resulted in a change in the ways they made sense of themselves
2. “The meaning and value of participating in school”: this reflects the role of the school in the learners' journey to recovery and adaptation post TBI. It includes views of how nonparticipation in school impacted on their recovery post TBI as well as how the school served as a vehicle for learners to progress post TBI
3. “Strategies used to adapt and resume participation in school”: this includes the cognitive-behavioural strategies that were used to support the resumption of school participation
4. “Journey of personal growth and perseverance”: overall, this theme reflects the positive personal attributes driving recovery and adaptation, the role of faith, the role of self-acceptance, and acceptance from others that assisted adolescents on their journey of personal growth and assisted them to persevere to full participation in valued occupations such as school
5. “Support needs for reentrance and participation in school post TBI”: for the purpose of this paper, this theme will be reported on (see Table 4)

This theme highlighted the need for support to increase the learner's capacity to make a positive school transition post TBI. The theme reveals that the network of support should ideally emanate from multiple levels within these adolescents' ecological systems (e.g., family, community, interdisciplinary teams, funders, and the school). Participants highlighted the need for therapeutic intervention to relearn old and learn new functions as a means to enhance day to day function. The need for effective communication channels as part of the planning for the learner's school reentry as well as the need for psychological support for both learners and their families was reiterated. A need for adequate resourcing within the Department of Education to provide the required learner support in ordinary schools and the effective implementation of applicable learner support strategies were identified as facilitators to school transition post TBI. Accessibility and safety within and around ordinary high schools as well as timely governmental financial support to the learner were indicated as needs in service provision that is aimed at supporting the school transition of learners post TBI.

#### 2.6.1. Enhancing Day-Day Function: Relearning Old and Learning New Function

Participants indicated that following the onset of the TBI adolescents experienced changes in their abilities, skills, and role fulfilment. In order to reengage in school, it required that adolescents relearn everyday function (i.e., walking, writing, and self-care skills) with the support from the interdisciplinary team on both an in- and outpatient basis. This is reflected by a learner and her mother:

I went to X Rehabilitation Centre to learn to walk (P5 Learner, line 16)

… at the beginning they (therapists) came there and they worked on him, they said, “We are working towards him to get better and to walk out of here and to go back into school” (P5 Mother, line 19)

Learners experienced changes in their mental functions, which impacted on their ability to complete school-related work activities. Mental functions refer to “functions of the brain, both global functions and specific mental functions” ([24], p.46). Whilst some learners received the therapeutic intervention to improve their mental functions, others did not. The lack of focus on mental functions as part of current interventions that are aimed at facilitating the school transition on post TBI was highlighted by a participant:

The one thing that I felt was missing from the rehab center, [was] enough focus on her mental functioning, it's not me criticizing what they do, it's just, there is a gap in what they offer… the biggest challenge thereafter was her mental growth (P2 Mother)

For other learners, the change in their level of functioning required that they learn new functional skills, such as personal coping strategies, to assist with their school transition. Personal coping strategies in this study relate to the cognitive and behavioural actions that learners put in place in response to adverse circumstances [25]:

We had to book him into a clinic where he could learn coping skills in order to adjust and to say it's ok to be like it, to fit in, you see (P6 Mother, line 126)

When asked what the experience at the clinic equipped him with, he responded:

Just things to keep calm and like it's ok to like need help sometimes because after a while I'm going to be fine again and I'm going to be able to do it (P6 learner, line 161)

#### 2.6.2. Effective and Open Channels of Communication as Part of Planning the School Transition

This category reflects the need for effective communication as a key element of the planning phase for learners' school reentry post TBI. This communication encompasses communication at two levels: firstly, communication between professional team members, learners, and their primary caregivers. This communication, amongst other aspects—participants mentioned, should include information on referral pathways and school options that would best suit learners' support needs post TBI. Most of the learners felt that there was no communication nor a formal process of preparing them for their return to school post TBI. Learners hence felt that they were not sufficiently prepared for the school reentry post TBI. This resonated with the views of their primary caregivers and the principals of the schools they attended. When asked how learners were prepared to reenter school post TBI, participants' responses were as follows:

No, I just went back to school (P4 Learner, line 29)

We just took him back to the school (P4 Grandmother, line 21)

Look, we from outside, we did not really work actively in preparing him; we have accepted him, but the preparation was done off our school, so I cannot really speak about the preparation (P4 Principal, line 134)

The second level of communication refers to intersectoral communication between the Departments of Health and Education regarding learners' abilities and needs to facilitate appropriate planning and structuring of the learner's support package. Participants reflected that the lack of intersectoral communication regarding a learner's abilities and needs post TBI resulted in teachers not fully understanding the learner's support needs:

The people at the hospital should tell the school more about brain injury (P1 Learner, line 120)

… it is something good if the doctor or whoever could have informed the school that they could have maybe helped him more in the school (P1 Mother, line 53)

I would have thought them to come to me, to give me a breakdown, or even to call the school to make an appointment to come… He's one learner out of 1300. How do you now give him specialized attention? We do not have that. So I would have expected that from the Health Fraternity or the Social Services; in this is how you handle it when there is a lapse of concentration, when he acts out… (Principal P1, line 18)

A lack of intersectoral communication regarding a learner's abilities and skills following the onset of the TBI also contributed to a mismatch in expectations:

Also for me when I went back, because I used to be quite academic, they put me in an academic class where everyone … it was the highest class with the smartest people in it, which maybe wasn't the best idea initially (P2 Learner, line 127)

#### 2.6.3. Learner Support Strategies

For all of the adolescent learners, the implementation of learner support strategies was needed to support their school transition post TBI. Learner support strategies include peer support and environmental, instructional, and assessment accommodations which influenced learners' capacity to reparticipate in school.

Most learners relied on their peers to support their initial school transition. Peer support included the use of peers' class notes, buddy systems for studying, and assistance from peers in areas where there were difficulties as a result of changes in functioning post TBI (e.g., hearing instructions or carrying a school bag between classrooms):

I took someone else's book; my friend's book in class so I can catch up with work (P4 Learner, line 71)

As a result of their changes in functioning post TBI, a few school environments provided accommodations to facilitate the learner's capacity to reparticipate in school. These environmental and instructional accommodations included extra classes, a graded return to school, preferential seating, and a reduction in the learner-teacher ratio. Some school environments attempted to grade learners' transition to school as is reflected by a learner and his mother:

I went for 3 subjects. They (those at school) gave us (learner and parents) a lot of leeway… three periods a day (P6 Learner, line 64)

She (the occupational therapist) wrote a motivation letter asking if they (the school) can assist with at least three subjects […] to find out how X's improvement will be and to make him school-ready for the following year; so they have accommodated that (P6 Mother, line 62)

Given changes in functioning (e.g., changes in mental and fine motor functions), a few learners were accommodated in terms of assessment practices. These assessment accommodations included the following: extra time for tests and exams, the use of scribes, simplification of test questions, and the use of open book tests. Of the few learners who were accommodated in terms of their assessment practices, most received extra time for tests and exams given their barriers to learning including changes in mental and fine-motor functions:

I used to get 10 minutes an hour extra which has changed to 15 minutes an hour, which I'm very thankful for because I need it (P2 Learner, line 70)

Yes, she got extra time. That was the one thing they did do, they gave her extra time (P2 Mother, line 35). She does write slower, you know, but the extra time helped (P2 Mother, line 44)

Most participants reported a lack of implementation of reasonable and effective learner accommodations within ordinary schools. This impacted on learners' school participation as well on their teachers' ability to provide the appropriate support:

There is such a high workload of seven subjects, it's very hard to keep up and I did nearly fail Grade 9, so it would have probably been better if I had just done some subjects at a time …instead of spread it over seven (P2 Learner, line 43)

She was very down. It was just that the workload was overwhelming (P2 Mother, line 50)

Getting scribes costs money. If X's parents could afford it, yes, we could get him a scribe (P5 Teacher, line 46-50)

#### 2.6.4. Adequate Resources within the Department of Education to Provide the Required Learner Support in Ordinary Schools

This category includes a need for resourcing from the Department of Education to increase the capacity of an ordinary school to provide learners with the necessary support. Human resources include a need for specialists at the departmental level (i.e., psychologists, social workers, occupational therapists, and other allied health professionals) who are able to deliver services to learners with moderate and high support needs. This is vital as without this support, it in turn impacts on the school's capacity to provide support to the learner:

Unfortunately being a mainstream school, we have to apply and there might be one psychologist for 20 schools. So 20 schools would translate to something like 20000 learners. So it becomes one in that 20000; we are losing it (P4 Principal, line 50)

Further human resources needed include sufficient staff to cater to varied learner needs within a classroom setting. Educators shared their perspectives on this:

I think also sometimes the schools are not equipped. We have so many learners and if you look at, I mean there is more than one child in a class that have issues, different issues. So how do you deal with all these children in one class? (P4 Teacher, line 100)

Learners, primary caregivers, teachers, and principals shared the sentiment that teachers require professional development/in-service training to enable them to understand the learners' needs and hence provide the learners with the appropriate support:

The teachers did not really understand what was wrong (P3 Learner, line 60)

I would say the teachers did not really understand, because they knew he was in an accident so they just let him be. So whenever he got a headache, after a time they could not send him home anymore, so they said “just sleep it off like in the class, just sleep it off… so for me it wasn't really up to standard (P3 Mother, line 62)

The teachers do not feel empowered. They want to, but they do not know how to and that is absolutely true and that's just because they are not trained. You know we expect schools to become inclusive and to become full-service schools, and it must just happen, but we forget that those teachers have not been trained (P3 Principal, line 35)

#### 2.6.5. Accessibility and Safety within and around Ordinary Schools

Participants mentioned that within ordinary schools there is a need for an increase in physical accessibility as current structural barriers (e.g., stairs and inaccessible toilets) impact on adolescents' ability to mobilize independently and safely in and around the school:

I go to a three-story school, so there are stairs everywhere, and I have classes on all three levels … (P6 Learner, lines 81-83)

physical barriers that are at X (referring to the high school where she teaches); so not having ramps, not having any staircases where kids can go up that are disabled, definitely that (P6 Teacher, line 105)

Accessibility could further be optimized through a change in the attitudes of those in the adolescent's school environment. Attitudinal barriers (e.g., negative attitudes of some teachers and peers as well as stigma) were mentioned as negatively impacting on the adolescent's school transition post TBI. Negative attitudes are reflected by some teachers and principals who expressed that at schools they are often so focused on completing the curriculum and learner throughput that there is often not sufficient time to spend with individual learners who experience specific barriers to learning:

Mainstream is geared towards positive pass, you need to pass, you need to stand out, the school needs to stand out, because if a school does not reach that 60% pass, they are being termed as a terrible school and immediately the following year, all the Departmental people are there. So principals are under the pressure to perform; they cannot sit still with that learner…It's very difficult in the mainstream because everyone is geared to CAPS (referring to the curriculum), there is pressure on learners; the principal wants his pound of flesh from every educator, is the syllabus done? (P1 Principal, lines 3 & 30)

A principal reflects that the negative attitude of some teachers regarding learners who require support as “being extra work” may also be a barrier to the reentry of these learners into ordinary schools:

... even our teachers view learners differently. Our opinion is formed before we start working with that child. This is now a nuisance, this is now a lot of work for me... (P4 principal, line 122)

The negative attitude of peers is also shared by some learners as a difficulty to their initial adjustment following their school transition post TBI:

The other children would say “she's mad”…then they just look at me with that intention, they do not even meet me or anything, talk to me … just no, she's mad (P7 Learner, line 81)

I was teased a lot… once I walked passed a girl in my grade and she said that I ran into a car – like she told her friends and they laughed at me while I was standing there with an eye patch. But I did not run into a car (P2 Learner, line 39).

A learner shares that the stigma in her community regarding special schools, is what contributes to her difficulty with adapting to this learner environment:

Also coming to school in the bus; I hate the word that says “Special School for Special Children with Special Needs”… people will think that I was born like this, but they will not accept I had an accident…Also another thing that the children and the people in my community, they put me under pressure in terms of you go to a school for physically disabled children, you go to a school for disabled children and then they like “so you, you're also disabled”. They make me feel bad for going to this school; they make me feel really bad (P8 Learner, lines 201-206).

A principal has the opinion that the stigma associated with brain injury may impact on the learner obtaining the needed support as it may result in the caregiver and the learner not making a full disclosure to the school regarding the extent of the changes in functioning post TBI and that this may pose some barriers to learning:

Sometimes they also have their reasons. You know at the high school, the name calling and things like that; the stigma that's attached (P4 Principal, line 106)

A need for safety for learners entering and exiting the school premises was highlighted as a support needed as a lack of safety impacted on the school's ability to provide learner support (e.g., extra classes) outside of school hours. The lack of safety further impacted on some learners' abilities to transition to extracurricular activities post TBI:

When we go home we used to go to the train station. It wasn't that safe, so we would all go as a group to the train station and climb on the train and each one gets dropped off like that (P3 Learner, line 158)

I stay in this area myself, but there are days that I fear for my children that go to school (P1 Mother, line 81).

But what you also need to take into account is the sub-cultures that exist outside of the school. Our communities are very territorial; so you belong to X (name of area) and that one belongs to X (name of area). So, during the school day it is fine, we cross those boundaries, but after school you come out of an area that's controlled by the ‘Americans' (local gang), and you move into an area that is controlled by the “Dollarbrands” then it becomes an issue…so these are all things that impact on schools after hours. We would so much want them to be integrated into sports, but we have got problems with presenting all these things (P4 Principal, line 71)

#### 2.6.6. Psychoemotional Support

Participants reflect that after a life-altering experience such as TBI, learners require psychological support. Participants highlighted the need for psychological support for learners to prepare and support them for the changes in functioning that may come to the fore upon discharge from the hospital/rehabilitation setting. This is needed to assist learners' acceptance of their change in functioning post TBI and to facilitate a positive adaptation:

They should speak more about …Because it's a lot to accept going home and needing help, so like speak to the patients before they go home and tell them “look here, when you go back home, things are going to be different and you're going to need a lot more help than what you needed previously” (P6 Learner, 190-192)

I think the only other extra thing you could have done, was mentally or emotionally, could have been the one, because I know he did get depressed at a certain time. That was after he was at X (rehabilitation centre) and they sent him home. From that time until he came back to school he was on another level depressed. He was sad, he was upset, he was cross with the world (P6 Teacher, line 52-56).

Learners, in particular, referred to the need for their support systems to foster a sense of agency, for example, through efforts at actively involving them in the planning of their school transition post TBI. A learner shared that his school reentry was a graded process in which he initially participated in three self-chosen subjects. This is reflected by his words:

I chose to go back to Life Sciences, Physics and Accounting (P6, Learner, line 100)

However, as the TBI also impacts on the family, primary caregivers emphasized a need for psychological support to extend to the immediate family of the learner with the TBI.

I think when one person in a family has an accident, it affects the whole family. So I think we all needed counselling, which I could not really afford. So I think that was something that we missed as a family because we were all battling (P2 Mother, line 88)

As a family we had zero support … she is the baby and she (referring to the learner's younger sister) saw what happened (referring to her son being shot in the head). The oldest one, he did not see it, but they were traumatized; we all were traumatized with it, but there was no one that came to us. We had to go and pay people to assist us as family, because it made an impact on our lives as well. We as parents, you know, what happened at the end, it affected my husband's work. It affected me when I went to work and he had to deal with me. It causes conflict as well.

Both the learners and their caregivers reported a need for psychological support in terms of planning for the future as the onset of the TBI meant that learners had to pursue alternate scholastic and ultimately future career goals. This adjustment for some learners and their families proved difficult leaving them with a sense of uncertainty about the learner's future:

I wanted to become a Scientist, and now I see science is not for me. I do not know what I want to be (P5 Learner, line 40 & line 140)

He's a teenager; whereto from there now? Where to with his life from there? … But he is now 16. We think ahead. When he is 18, what is there for him to replace, because now my husband says what are we going to do; where do we put him? He is not a child that can go to a normal Technicon (P5 Mother, lines 190-191)

Learners highlighted that their transition to school post TBI required emotional support from all involved. In particular, primary caregivers were mentioned as a key source of emotional support, as they often served as the advocator for their child to return to school as well as fulfilling a role as an intermediatory between the Departments of Health and Education:

My parents contacted the school (P5 Learner, line 82)

We did make the contact with the school. My husband and me went there and speak to the principal… And you know the amazing part of his father, he never stopped. He never stopped at anything. He went to the highest of the highest of the highest of all to get our child to what he is today, and that he saw, that his parents are fighting for him; just not leave it there; we fight for him (P5 Mother, line 157)

The parents are playing more than their role on his side (P5 Teacher, line 32)

Learners also highlighted other important sources of support, i.e., family, friends/peers, and teachers, as well as the support from the broader community, specifically religious organizations to which some families had strong connections to. This acceptance from others, their reassurance, and encouragement were instrumental in assisting with the learners' school transition:

My family played the supporting role. It's a role that they like never gave up on me (P3 Learner, line 144).

It is the top class that he is in and that is where his motivation comes from… His friends that he has, are top learners (P1 Teacher, line 38)

We wanted her to integrate back in her social environment and I think all the way through Grade 9 – because she came back for Grade 9 – we actually just let things ride. If she was going to get through, she was going to get through and if she did not, we would take those decisions when it got to that point. But more a case of allowing her to believe in herself again, which was a very slow process for her (P2 Principal, line 29)

I mean a lot of people prayed. I mean we had Priests over the world praying. (P6 Learner, lines 171)

Both learners and their primary caregivers highlighted the need for emotional support given the impact of the TBI on the learner's interpersonal interactions. The adjustment to the changed life circumstances post TBI often resulted in strained relationships with a learner's main support system (i.e., family) and further resulted in decreased social interaction and the loss of friendships:

My friend group that I had before, stopped being my friends and I was kind of alone (P2 Learner, line 6)

She lost all her friends… and she did not know how to make friends. (P2 Mother, line 39)

#### 2.6.7. Timeous Financial Assistance from the Government

Primary caregivers mentioned the socioeconomic implications of the untimely financial assistance from the government (i.e., Road Accident Fund or Disability Child Grant) to assist with the coverage of their children's learner support needs:

Finance is a big part of it. If the Road Accident Fund actually ever does pay you out, you have to wait 5 years, but in the first 5 years, it is when you need it most. That's your optimum time for brain injury recovery and that's when you need the therapy, when you need the support, when you need the training, it's at that point. After the 5 years it's almost … you missed the boat (P2 Mother, line 88)

In most cases, the primary caregivers were the primary financiers of the service provision that learners required post TBI (either out-of-pocket or through the payment of private health insurance coverage). Participants hence reiterated the financial constraints that served as a challenge for the learners with TBI to be provided with the needed support:

He came out of hospital, he gets every day physio, every day therapy; we cannot go every day to X, (referring to state hospital) we do not have that money (P5 Mother, line 187)

Getting scribes costs money. If X parents could afford it, yes, they could get him a scribe, but we struggle as a school just to make ends meet at the end of the month, just to pay our …. I'm on the Governing Body, so I know these things. Our account is in the minus, so we do not have resources like that (P5 Teacher, line 50)

## 3. Discussion

This paper highlighted support as a key theme that emerged from the findings of the study. The findings provide insight into the support needs of adolescent learners' school reentry and school participation post TBI within a developing context. The discussion will expand on these support needs in terms of the stage, level, and type of support needed to facilitate high school participation post TBI. In an endeavor to allow for an increase in the level of responsiveness of occupational therapy services to these learners' support needs, the authors seek to discuss the potential role of occupational therapy in school transition practice post TBI within a developing context.

Similar, to the studies by Sharp et al. [7] and Mealings and Douglas [5], participants highlighted that they felt that learners need support at various stages of school transition post TBI. This firstly includes a preparatory stage for the learner's school transition given the changes in their functioning post TBI. The second one is transitionary support as the learner reenters and participates in school (i.e., during the initial period of adjustment for both the learner and those who provide the needed support). Mealings and Douglas [5] in addition highlight the need for a third stage of support during the grade to grade transition specifically as barriers to learning may become more pronounced as the scholastic demands increase. Participants in this study reveal that current support is lacking in terms of preparing learners with TBI for the transition post high school. This concurs with existing literature which iterates the need for a stage of transition planning for post high school as a key to assisting learners with TBI and their families to put measures in place that identify and plan for the attainment of their vocational goals [5, 15, 26, 27].

Participants further alluded to support that should come from different systems within the learner's context. Using Bronfenbrenner's bioecological systems theory [28], these interrelated support systems may be viewed as those that span to include the microsystem (for example, the support that the learner receives directly from their teachers), mesosystem (for example, the support that the teachers and parents receive from each other which then indirectly impacts on the learner), and a macrolevel (for example, the government ensuring that the applicable inclusive educational policies are adequately resourced to allow for the adequate implementation thereof). Within the chronosystem, the findings of this study also revealed that although inclusive education had been implemented and policies such as the Education White Paper 6: Special Needs Education-Building on Inclusive Education and Training System [29] and SIAS [10] provide guidance on inclusive education provisioning in South Africa, schools have not been prepared for the changes needed in the education system over time. This is evidenced by study participants' reports of the following:

- Structural barriers within schools
- Teachers reporting a lack of technical equipment needed to provide alternative instruction and assessment
- Teachers reporting inadequate training on inclusive education and the relevant policy
- Learners being inadequately supported by structures within the school and the district

The types of support that were explicated above as fundamental in support provision to optimize an adolescent's school transition post TBI allude to the need for the occupational therapist to fulfil various roles as part of the school transition team. These roles may include that of a facilitator, intermediary, coach, collaborator, supporter, and advocator. The tasks that underpin these roles may contribute to the school transition, either directly or indirectly (i.e., support to other members of the team—notably the adolescent's family and teachers) [30].

Learners in this and other studies have reported that post TBI they experienced changes in abilities, skills, and role fulfilment [5–7]. These changes often impacted negatively on their self-esteem, motivation, and competence in their day to day functioning. This in turn negatively impacted on their sense of belief in self and on their ability to transition to various life situations post TBI including that of school [31]. As a *facilitator*, the occupational therapist may cultivate the adolescent's belief in self through facilitating the adolescent's skill development and experiences of mastery through enabling the adolescent to identify and employ remediative and compensatory strategies that allow for the engagement in academic, nonacademic, and extracurricular school-related activities. As part of this input, the occupational therapist should facilitate goal setting, regarding the adolescent's prioritization of abilities and skills to be relearnt and compensatory strategies that could be put in place. Adolescents should further actively partake in planning their school reentry as well as be involved in the development and monitoring of action plans to support their participation in school. Cultivating such opportunities for the adolescent to display agency in negotiating the resources they require to meet their support needs is thought to link to positive developmental/participatory outcomes of youth [32, 33].

Most of the learners reported that they were insufficiently prepared for their return to school following being discharged from the hospital or the rehabilitation unit. This resulted in a perceived lack of competence and confidence and affected their school participation. The occupational therapist could facilitate the personal preparation of the learner through input such as the following:

- Incorporating school-related activities as part of therapeutic interventions in a hospital or rehabilitation setting to prepare the adolescent to assume the learner role
- Social preparation with assent and consent from the learner and family facilitating brief visits with a few of the learners' friends/peers (in hospital or once discharged) to provide the learner opportunities for social reengagement prior to the learner's return to school
- Preparing the learner how to answer questions regarding TBI
- Preparing the learner how, what, and when to disclose information about TBI
- Graded return to school including a brief visit to the learner's homeroom class and attendance of school for a few hours/learner attending specific subjects, building up to a full day. Learners should have input with regard to the subjects they would like to complete as part of their graded return to school. Subject choice should however match the learner's strengths to allow him/her the opportunity to achieve mastery and rebuild confidence [12]

Positive interactions between adolescents and those in their context could also be fostered through open and clear channels of communication between all key role players in the school transition [34]. Similar to the findings in other studies, the need for a formal link between health and educational teams was similarly shared by participants in this study. Learners reported that the lack of communication from the health team meant that teachers were unable to implement the applicable accommodations needed to optimize the learner's school participation [34, 35]. The occupational therapist could therefore fulfil the role of the *intermediary* between the health team and educators in the adolescent's school context regarding the effect of TBI on the adolescent's functioning, needs, and strengths. This may enable an increased understanding of a learner's support needs and result in realistic expectations of the adolescent. This may further assist the adolescent to feel understood and accepted and experience a sense of connectivity which learners in this study highlighted as a facilitator to their school transition post TBI. Information exchanges should strive to highlight the strengths and potential of the adolescent to cultivate positive attitudes towards who the adolescent is post TBI. Information exchanges should also seek to clearly define the roles of all team players, and discussion should include a preliminary plan of action regarding the learner's return to school. The discussion could include the following: the best fit of a school environment given the level and extent of the learner's support needs, the process of the school return (graded or complete return), a decision regarding the timing of the school return, the need for a school visit to assess the school environment, and possible strategies that could be put in place to support the learner's school participation.

Learners in this study reiterated the role of external support from those in their context in terms of increasing their capacity to make a positive transition post TBI. In this study, the external supports (including primary caregivers) mentioned that they often felt that they did not have the capacity to provide the adolescent with the needed support. Occupational therapists could therefore serve as *coaches* in which they build the resources of the adolescent's support base, specifically those who serve as informal sources of support. Informal supports are sources of psychosocial support that are “lay, spontaneous, and often based on reciprocal obligation” ([36], p.39). It includes family, friends, and fellow members of faith-based, recreational, or community organizations. Within a developing context such as South Africa, formal supports (e.g., professionals) tend to have more short-term relationships with adolescents given high caseloads and limited funding. Building the resources of informal supports notably that of a family is of importance, specifically as they tend to play an active and ongoing part of the adolescent's life and are therefore potential long-term consistent sources of support [36]. *Coaching* includes knowledge exchange regarding the adolescent as well as on specific strategies that can be employed to support the adolescent's participation in occupation [37]. For example, the family could be educated to employ adaptive strategies where they are taught to (a) modify the task (e.g., simplifying tasks by changing the steps of the activity) and (b) modify the environment by minimizing or changing environmental factors: noise, level of activity, etc., that may impact on the adolescent's performance. In keeping with a client/learner-centered approach, the strategies that will be employed need to be developed in collaboration with the adolescent. During this communication, it is crucial that primary caregivers are seen as part of the team and involved in the assessment and planning of their child's developmental plan given their intimate knowledge of their children's strengths and weaknesses as well as their functioning [37]. It is important that primary caregivers are continuously regarded as active partners throughout their child's schooling, through them receiving regular feedback regarding the monitoring of their child's progress as well as their involvement in their child's post school planning.

Occupational therapists may further build relationships with relevant school personnel to *collaborate* in the planning of support provision. Collaboration with the teachers and other relevant school personnel on professional development initiatives that seek to build on teachers' knowledge and skills on specific adaptive strategies can facilitate efficacy and confidence and fosters positive attitudes amongst teachers [38, 39]. When teachers display positive attitudes (e.g., display openness to work with the adolescent following their changes in functioning and to adapt their teaching practices), positive teacher-adolescent interactions are fostered [5]. Therefore, a collaboration should include a teacher who actively teaches the adolescent and is hence able to use their knowledge and experience of working with the adolescent to inform input on strategies. The teacher should ideally also be the one who will be responsible for the implementation and monitoring of the individual support plan. However, as this teacher may change as the learner progresses from grade to grade, it is recommended that a more constant educator may also be involved (e.g., guidance teacher or member of the school-based support team). Information exchanges with teachers should be facilitated in a manner that seeks to acknowledge and expand on their existing knowledge [37].

Psychoemotional support is important as the school transition is likely to occur during a time when the learner is undergoing a period of adaptation and reconstruction of self, following changes in functioning and in their former sense of self [40]. Learners referred to difficulties they experienced with the school transition given the changes in functioning and levels of dependence and for some drastic changes in appearance post TBI. Psychological support they alluded to was needed to equip them with the necessary coping strategies amongst others dealing with the expectations from parents, educators, and peers for them to be functioning at the same level as they were prior to the injury. Psychological support was therefore required in the preparatory phase as well as during the first year of being back at school. This was seen as vital in assisting learners to monitor and adapt coping strategies as well as for them to address emotional challenges they experienced once being back at school. The occupational therapist as a *supporter* provides emotional support through active listening, being empathetic, and providing encouragement [37]. Emotional support promotes positive interactions between the adolescent and significant others in their environment. This is important given the impact of the TBI on the adolescent's emotional functions that may affect their interpersonal interactions and relationships, specifically with those whom they rely on for support [14, 31, 41, 42]. As primary caregivers in this study highlighted that TBI also has an impact on the family unit, it is imperative that emotional support should be provided to the family of the adolescent with TBI. Emotional support is needed to build the family members' coping resources which will enable them to provide support to the adolescent [37].

As an *advocator*, an occupational therapist should advocate that all team members foster the attitude of viewing the adolescent with TBI as capable and having potential (i.e., use a strength-based approach). Furthermore, the occupational therapist may empower (a) the adolescent's informal support base (i.e., family) to advocate and (b) the adolescent to self-advocate for the right to inclusive school participation to be upheld through negotiating for the availability and access to meaningful and culturally relevant resources. The need to build self-advocacy is supported by Mealings, et al. ([40], p.10), as it positively influences the adolescent's self-esteem and self-reliance and enhances their opportunities to seek appropriate support. The therapist develops the adolescent's self-advocacy skills through education on their right to a quality education as well as the range of support that should be made available and accessible. Education is done using the adolescents' frame of reference and developing the adolescent's assertiveness and communication skills. Such skills are necessary to increase the adolescent's capacity to seek and ask for the learner support they require. In addition, the adolescents' families should be educated on the adolescent's right to inclusively participate in valued occupations such as school as well as their right as parents to be actively involved in decisions pertaining to their child's schooling. Information regarding what this right entails as well as the availability and access to resources to support their child's needs as outlined in the applicable South African Legislation on Inclusive Education should be clearly communicated as a basis for developing their advocacy skills.

### 3.1. Recommendations for Future Research

The current study could be expanded on to include the perspectives of participants within rural contexts in South Africa as well as in other developing contexts. This is needed to explore if there are rural-urban differences in the support needs of learners in terms facilitating their school transitions post TBI. This would also increase the generalizability of the findings.

A longitudinal study could be conducted, where the participants in this study are followed up to explore their support needs to prepare for their post high school transition.

### 3.2. Limitations of the Study

The study population did not include adolescents with severe language and cognitive difficulties. The sample size of eight cases is small, but it should be reiterated that the purpose of qualitative research is to gain an increased understanding rather than to generalize results. The participants recruited for this study did not allow for variation in the sample in terms of geographical setting (i.e., rural vs. urban) or much variation in terms of culture. Including adolescents who come from rural areas may have allowed for an increased insight into the role that the context, specifically in terms of access and availability of support services, plays in their experience of school reentry and participation. Adolescents with diverse cultural backgrounds may have also provided an increased understanding of the influence of culture on school transition following a newly acquired disability such as TBI. It should however be noted that in South Africa, there is no central database with information regarding learners' barriers to learning or categories of disability. This impacted on the recruitment of participants and variation in terms of the sample.

## 4. Conclusion

This study highlights the support needs for optimal school transition post TBI. It seeks to outline the diverse role of the occupational therapist as a member of the school transition team, in response to these needs. Findings reiterate the need for school transition services post TBI to include support that seeks to include personal preparation of the learner as well as preparation of the learning context. Ultimately, high school transition post TBI requires a community of support that is characterized by the active involvement of learners and their primary caregivers, where the roles of all key role players are clearly defined and there are open and clear channels of ongoing communication.

## Figures and Tables

**Table 1:** Selection criteria for the study participants.

Inclusion criteria	Exclusion criteria
Adolescent learners (i) Participants within middle and late adolescence (13 to 20 years of age) (ii) Participants with an onset of TBI within adolescence (iii) Participants with stage 3 mild, moderate, and severe TBI. The severity of the TBI was obtained from the participants' medical records (iv) Participants that resided in four urban districts and two rural districts in the Western Cape, South Africa (v) Participants who have had intervention by a medical doctor and rehabilitation by member(s) of a multidisciplinary team (vi) Participants who have reentered school (ordinary or special schools) for at least three months post TBI (vii) Participants who have attended an ordinary secondary school prior to the onset of the TBI and not have required specialist learning support during their prior school history (viii) Participants from any racial group to account for cultural and ethnic diversity but who had to be able to understand questions and communicate in English, Afrikaans, or isiXhosa (the three predominant languages spoken in the Western Cape Province)	(i) Participants with severe cognitive and language impairments post TBI (ii) Participants with premorbid psychiatric conditions
Primary caregivers (i) Participants resided in the above-mentioned districts of the Western Cape (ii) Participants were the primary person caring for the learner (iii) Participants had to be able to communicate in English, Afrikaans, or isiXhosa	
Teachers and principals (i) Participants were based at the ordinary school that the learner returned to post TBI or were based at the special school that the learner was transferred to post TBI (ii) Participants had to be able to communicate in English, Afrikaans, or isiXhosa (In addition, a teacher is needed to be identified by the learner as knowing him/her the best and who was part of the high school transition)	

**Table 2:** Demographic description of participants.

Participant	Age	Gender	Grade	Type of school at time of interview	Age at onset of TBI	Severity of TBI	Language	Socioeconomic status
Learner 1	17	Male	11	Ordinary	16	Mild	Afrikaans	Low
Learner 2	19	Female	12	Ordinary	15	Severe	English	Middle
Learner 3	15	Male	9	SS	14	Moderate	English	Low
Learner 4	15	Male	9	Ordinary	14	Moderate	Afrikaans	Low
Learner 5	16	Male	8	SS	15	Severe	English	Low
Learner 6	18	Male	12	Ordinary	17	Severe	English	Middle
Learner 7	20	Female	Left school in grade 10	Ceased schooling	15	Severe	English	Low
Learner 8	16	Female	10	SS	15	Severe	isiXhosa/English	Low

SS = special school.

**Table 3:** Data analysis (case by case analysis and cross-case analysis).

Step 1	Preparation of the data (i) Verbatim transcription of interview scripts (ii) Typed hand-written field work notes onto a MS Word document (iii) Created memos in ATLAS.ti, version 7 (qualitative analysis software programme) (iv) Typed excerpts from medical and school records onto a word document

Step 2	Organizing the case study database (i) Uploaded and organized data from interview transcripts, field notes, and relevant documents onto ATLAS.ti, version 7

Step 3	Read an interview transcript, field notes, and document collected (e.g., interview transcript of learner 1, field notes of interview with learner 1, and the medical/school records of learner 1)

Step 4	Open coding (i) Took the interview transcript of learner 1 in case 1 and using ATLAS.ti, version 7 highlighted segments of data that were potentially relevant to answering the research question and added codes and comments

Step 5	Axial coding (i) After working through the entire transcript of learner 1, a code list was printed and manually checked for duplications, and codes were renamed, combined, or deleted (ii) The codes were copied and pasted onto a word document (iii) Grouped codes together that appeared to link, forming categories (iv) A separate running list of categories was kept

Step 6	Repeated steps 3-5 with the remaining sets of data in case 1 (i.e., interview transcripts and field notes of interviews with primary caregiver, teacher, and principal) whilst keeping in mind the previous lists of categories checking whether they were also present in the subsequent sets of data

Step 7	The lists of categories of all the data sets in case 1 were compared

Step 8	All the categories of all data sets in case 1 were merged onto one master list of categories Those categories that held up across sets of data for case 1 were retained, some categories became subcategories, and others were subsumed. Categories were hence refined throughout the process of analysis. This list reflected the recurring regularities/patterns in the study that cut across all sets of data of case 1

Step 9	Steps 1-8 were repeated for each of the remaining cases (i.e., cases 2-8)

Step 10	Cross-case analysis (i) Each case's main categories were examined to explore the similarities and differences amongst individuals and contexts. This assisted to determine shared factors that influence school reentry and participation and propose personal/contextual features that explain variations (ii) Through a process of comparing and merging salient categories, the subcategories and categories that were consistent across cases were then grouped into themes resulting in a unified description of categories and themes across cases

**Table 4:** Support needs for reentrance and participation in school post TBI.

Categories	Subcategories
(i) Enhancing day-day function: relearning old and learning new functions	(i) Relearning old function (ii) Relearning new function

(i) Effective communication channels as part of the planning for school reentry	(i) Effective communication between other team members, learners and their primary care-givers impacting on learners' preparedness for school reentry (ii) Intersectoral communication between the Departments of Health and Education regarding learners' abilities and needs

(i) Learner support strategies	(i) Peer support (ii) Effective implementation of environmental and instructional accommodations (iii) Effective implementation of assessment accommodations

(i) Adequate resources within the Department of Education to provide the required learner support in ordinary schools	(i) Adequate human resources to provide specialized learner support (ii) Adequate training of educators in ordinary schools on relevant policy and learning support

(i) Accessibility and safety within ordinary schools	(i) Physical access (ii) Limiting attitudinal barriers (iii) Safety within and around ordinary schools

(i) Psychoemotional support to both the learner and family	(i) Psychoemotional support to the learner (ii) Psychoemotional support to the family

(i) Timeous financial assistance from the government	(i) Limit financial constraints that hamper learner access to support (ii) Limit financial implications on household for out of pocket expenditure to support the learner's needs

## Data Availability

The interview data used to support the findings of this study are restricted by the Human Research Ethics Committee, Stellenbosch University in order to protect participant privacy.

## Supplementary Materials

Supplementary Materials Appendix A: example of interview guide for learners with TBI.
