# Supplementary material for: A Service User Perspective Informing the Role of Occupational Therapy in School Transition Practice for High School Learners with TBI: An African Perspective
Source: Occup Ther Int. 2019 Aug 1;2019:1201689. doi: 10.1155/2019/1201689 (PMC6701279; doi:10.1155/2019/1201689)
Supplement: Supplementary Materials — Appendix A: example of interview guide for learners with TBI. [file 1201689.f1.pdf]

Appendix A: Example of interview guide for learners with TBI

- Tell me about how you first felt about going back to school after your brain injury?
- Tell me when did the planning for your return to school first start?
- Was there a meeting to talk about you going back to school?
- Tell me about how you were prepared for your return to school?
- Do you think you were prepared enough for your return to school?
- Do you think that the people at school (i.e. teachers, peers, principal, other team members) were prepared for your return to school?
- What do you think could have been done differently or extra to help you be better prepared for when you returned to school?
- Tell me about when you first came back to school after your injury, how did that go?
- What did the school put in place to help you cope at school?
- Some people would say that when returning to school after a traumatic brain injury there may be some challenges. What would you tell them?"
- Who helps you with your school work at home?
- What do you think could be done differently or extra to help you do well at school?
- What does going to school mean for you?
